# How auditory development affects language acquisition: Influences of socioeconomic status and gestational age at birth

**DOI:** 10.1371/journal.pone.0341841

**Published:** 2026-02-05

**Authors:** Susan Nittrouer, Dalton Burchardt, Aaron McEnery, Joseph Antonelli, Ruchita Kachru, Juan C. Roig, Thomas Schrepfer

**Affiliations:** 1 Speech, Language, and Hearing Sciences, University of Florida, Gainesville, Florida, United States of America; 2 Department of Statistics, University of Florida, Gainesville, Florida, United States of America; 3 Pediatrics, University of Florida, Gainesville, Florida, United States of America; 4 Otolaryngology – Head & Neck Surgery, University of Florida, Gainesville, Florida, United States of America; Max-Planck-Institut fur Kognitions- und Neurowissenschaften, GERMANY

## Abstract

Technological advances in recent decades have intensified the need for strong language and literacy skills, such that deficits in these skills can significantly reduce occupational opportunities and social richness. Nonetheless, the causes of language and literacy deficits remain scarcely understood, so treatment consists mostly of drill on the very skills affected individuals struggle to perform. The purpose of this study was to test two related hypotheses. Hypothesis 1 was that delays in the development of the central auditory pathways greatly constrain acquisition of language skills dependent upon auditory development; these skills primarily involve those that are late emerging, namely phonological sensitivity. A corollary is that language skills that begin emergence early in life are less affected by delays in auditory development; this largely encompasses lexicosyntactic knowledge. Hypothesis 2 was that some conditions heretofore recognized as impacting language acquisition (poverty and premature birth, for the purpose of this study) take their toll at least in part by constraining the timely development of the central auditory pathways. To test these hypotheses, 104 children (5–6 years old) spanning continua of socioeconomic status and gestational age at birth were tested on (1) three measures of suprathreshold auditory functions associated with development of the central auditory pathways, (2) two measures of lexicosyntactic knowledge, and (3) two measures of phonological sensitivity. Results largely supported both hypotheses: Strong relationships were found between suprathreshold auditory functions and language measures, especially phonological sensitivity, and both socioeconomic status and gestational age appeared to exert their influence on language acquisition completely or partly through an effect on auditory function. These results should serve to refocus the search for causes of language and literacy deficits from purely environmental shortcomings to biological determinants, with newly inspired directions for interventional approaches.

## Introduction

### Problem statement

It is widely accepted that language is a uniquely human capacity. Members of other species communicate, but those communications are not as complex as human language, in either acoustic or linguistic structure. In spite of its greater complexity, however, human language necessarily evolved within the constraints of the mammalian auditory system, a system shared by all mammals. For individual children, spoken language must develop within the constraints of their own auditory capacities, but these capacities themselves change across childhood, so the constraints imposed on language development by those auditory capacities are ever changing. Thus, we may predict that if the development of those auditory functions is delayed, language acquisition will be delayed, as well. The investigation reported here was undertaken to explore hypotheses arising from that prediction. Specifically, these are: *(Hypothesis #1) The timely development of auditory functions more strongly affects later-emerging language phenomena, such as sensitivity to phonological structure, than language phenomena that begin to emerge early, such as lexical and syntactic (i.e., lexicosyntactic) knowledge.* Lexical, syntactic, and phonological knowledge were examined to test this hypothesis. *(Hypothesis #2) Some conditions known to negatively influence language development actually exert that influence by interfering with the development of auditory functions.* Two conditions associated with delayed or disordered language acquisition were invoked in this investigation as a way of exploring this second hypothesis; these were low socioeconomic status and premature birth. Although these conditions are often defined with somewhat arbitrary cut points (e.g., premature birth may be defined as anything less than 36 weeks gestation), we used continuous variables in this work based on the premise that the magnitude of effect should vary in a continuous manner. Including children along the continua of socioeconomic status and gestational age at birth also served to enhance variability in auditory and language measures, thus contributing to the successful testing of Hypothesis #1.

### Auditory development and language acquisition

**Fig 1:**
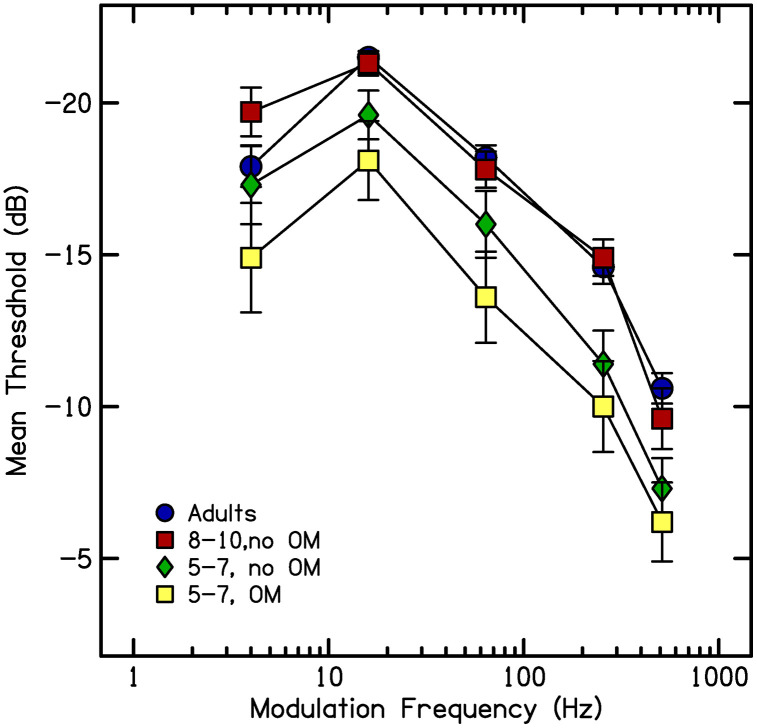
Modulation detection thresholds for temporal modulation at five rates for four groups of listeners (from Nittrouer and Lowenstein [8]).

**Fig 2:**
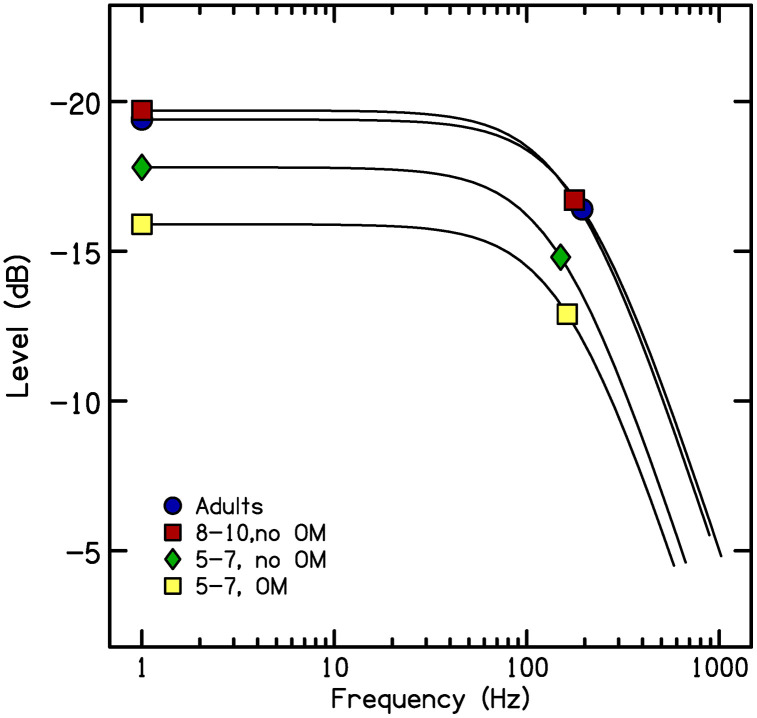
Temporal modulation transfer functions derived from thresholds shown inFig 1. Symbols on the left are the dB values at intercept and symbols on the right are the frequency boundaries (in Hz) of resolution, indicated by the 3-dB down points.

A newborn infant has auditory capacities that differ in many ways from those of adults: auditory thresholds are higher [1], frequency resolution is poorer [2, 3], and sensitivity to temporal modulation is poorer [4, 5], to name a few. Some of these functions quickly reach mature status; primarily those that rely on peripheral mechanisms [6]. But the development of other auditory capacities extends to puberty; these are functions involving the central auditory pathways. As an example of developmental effects in auditory capacities, Hall and Grose [7] obtained *temporal modulation transfer functions* for children between the ages of 4 and 10 years, as well as for adults. Transfer functions are computed from thresholds marking how deep the modulation needs to be at various rates of modulation for the listener to detect that modulation. To obtain these thresholds, investigators present stimuli consisting of noise modulated in amplitude across time by sinusoidally shaped envelopes. The depth of valleys in those envelopes is varied adaptively until an estimate is obtained of how deep the modulation needs to be for the listener to detect it. Thresholds are measured at varying rates of modulation from relatively low (e.g., 4 Hz) to quite high (e.g., 512 Hz). Fig 1 from Nittrouer and Lowenstein [8] illustrates mean thresholds for four groups of listeners across five modulation frequencies. These thresholds can be used to compute transfer functions, as shown in Fig 2, which displays temporal modulation transfer functions for the mean thresholds from the four groups with data shown in Fig 1. The height of the function at the intercept (in dB) is considered a metric of the listener’s sensitivity to this modulation; those values are indicated by the symbols at the left of the functions in Fig 2. The point where the function drops below 3 dB of that intercept (in Hz) serves as a metric of the resolution of the listener’s auditory system, meaning how tightly spaced the modulation can be for the listener to detect it; those values are indicated by the symbols towards the right in Fig 2. Resolution, largely a property of the peripheral auditory system, has consistently been found to reach adult levels early in development, certainly before four years of age [7, 9]. This finding is illustrated in Fig 2 by the fact that the 3-dB down point is similar (in frequency) across groups. Sensitivity to modulation, on the other hand, does not achieve mature status until roughly ten years of age – for children developing in typical fashion. This finding was reported by Hall and Grose, and is illustrated in Fig 2 by similarity (in dB) of intercept for the oldest children (8- to 10-year-olds) and the adults, but poorer (less negative) thresholds for 5- to 7-year-old children with no histories of chronic otitis media. Finally, these figures illustrate that 5- to 7-year-olds with histories of chronic otitis media (i.e., six or more episodes before age 3 years, as diagnosed by an otolaryngologist) had poorer sensitivity than age-matched peers without those histories. In the most severe cases, otitis media can temporarily raise auditory thresholds. As seen in Figs 1 and 2, this is one condition that can disrupt the timely development of at least one auditory function, sensitivity to temporal modulation. The current study examined two other conditions that could potentially have the same disruptive effects.

**Fig 3:**
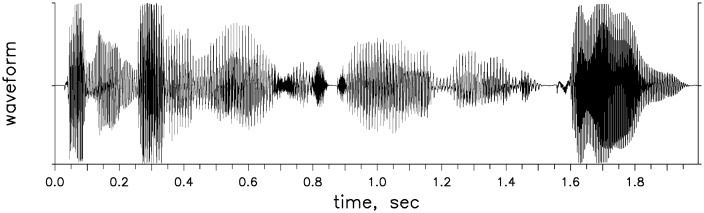
Waveform of a sentence, illustrating temporal modulation inherent in speech signals.

Fig 3 displays a speech waveform and offers insight into what this late development of sensitivity to temporal modulation could mean for language development. As can be seen, the continuous speech signal is amplitude modulated across time, with depth of modulation varying from very deep to relatively shallow. It is reasonable to suggest that the more sensitive a child is to temporal modulation, the better that child will be able to use patterns of amplitude change in the speech signal to recover linguistic structure. The fact that mature sensitivity to temporal modulation is not reached until 9 or 10 years of age, even for children developing on a typical timetable, means that younger children are processing speech signals without access to the same level of detail in these modulation patterns as adults and older children. Release from that constraint is delayed for children with delayed auditory development, such as the 5- to 7-year-olds with histories of otitis media whose data are displayed on Figs 1 and 2.

**Fig 4:**
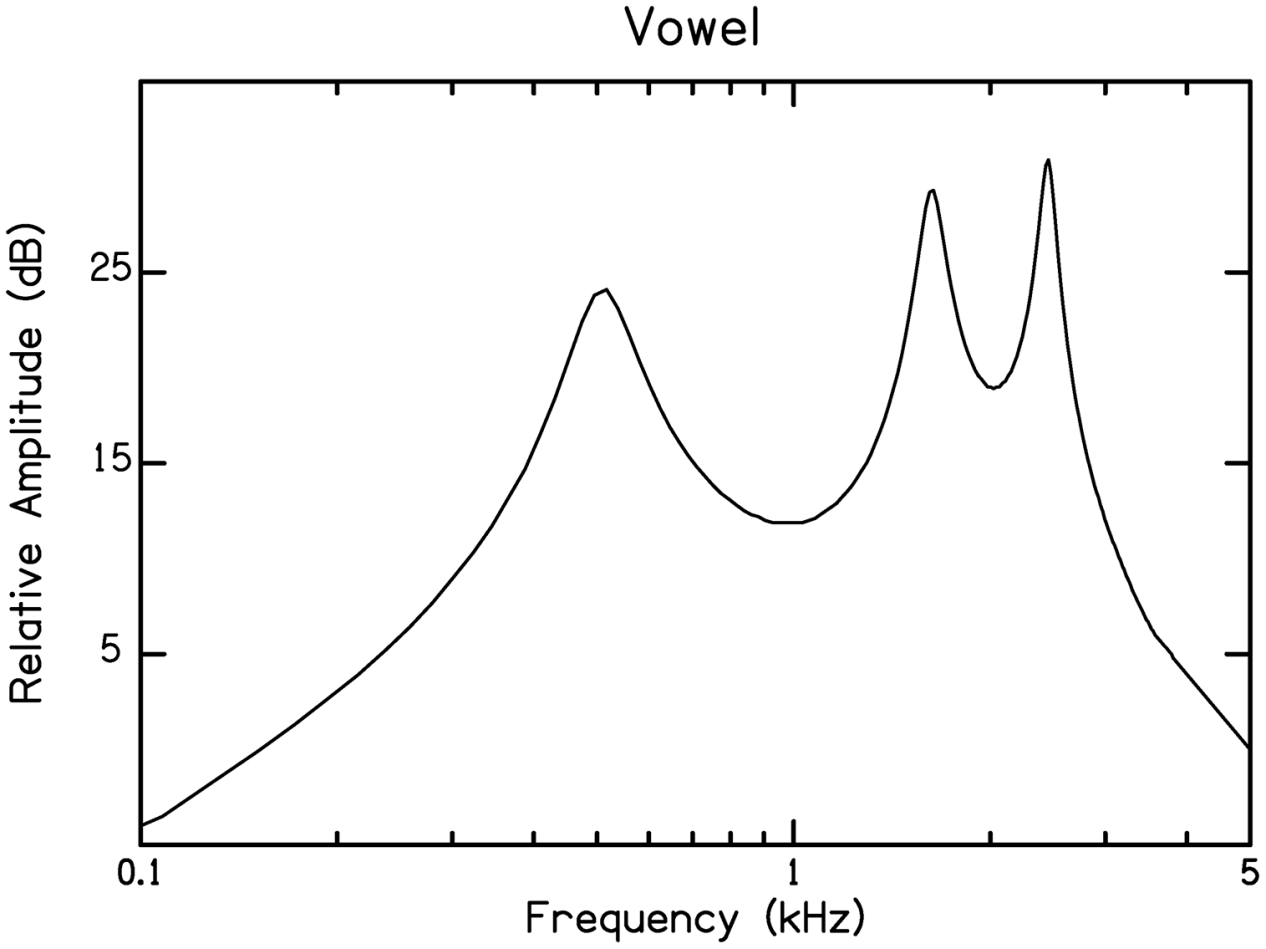
Spectrum of a vowel, illustrating spectral modulation inherent in speech signals.

A similar situation exists for the development of sensitivity to spectral modulation. This modulation refers to the pattern of change in amplitude across the spectrum. As with temporal modulation, it is possible to separately examine resolution, meaning how tightly spaced spectral peaks in that envelope can be and still be distinguishable, and sensitivity, meaning how deep that modulation needs to be to be detected. And as with temporal modulation, resolution is dependent largely on maturation at the level of the cochlea and auditory nerve, a function that reaches maturity very early in life, likely in the first year [3,10]. Sensitivity to spectral modulation, on the other hand, emerges only gradually across childhood [11–14]. To measure this sensitivity, sinusoidally shaped envelopes are imposed on a signal with an otherwise flat spectrum. Unlike stimuli used to assess sensitivity to temporal envelopes, the stimulus remains constant in shape across time. The depth of these envelopes is varied adaptively to obtain thresholds. The rate of modulation can be manipulated experimentally, but low rates of modulation, around 0.5 or 1.0 cycles per octave (cpo), best match the rate of modulation in the speech signal. This fact is illustrated in Fig 4, showing the spectrum for a vowel. In speech signals, this modulation forms the formants, and they occur at a rate of slightly less than one per octave. Again, it is reasonable to suggest that keener sensitivity to this modulation will better support recognition of the speech signal, especially phonemic structure. Sensitivity to spectral modulation, however, reaches maturity even later than sensitivity to temporal modulation [12], leaving young children unable to fully access this modulation in the speech signal.

### Beginning units

The initial unit of linguistic organization for the child is different from what the adult may use; for very young children, it is something resembling the whole word [15–19]. When infants enter the world, they have no pre-existing linguistic representations. A network of such representations must be assembled through the child’s interactions with the language in the environment, which is available only in the form of continuous speech. Thus, the very first task facing the child is to develop a strategy for extracting meaningful units from that continuous signal [16,18,20]. To be sure, the infant has several tools available to support this endeavor. First is the fact that the units most meaningful to the child will likely be those that are repeated most often, such as the child’s name or labels of objects relevant to the child’s life. Hearing a consistent acoustic pattern in conjunction with the presence of a specific object will facilitate the extraction of that particular stretch of acoustic signal and its subsequent establishment as a discrete lexical representation: e.g., the acoustic pattern comprising the word *doggie* consistently occurs when the family dog is present, albeit in different linguistic contexts. Other perceptual tools are available for this initial word learning, as well. For example, infants quickly learn to use language-specific stress patterns to isolate words [21]. Nonetheless, in the early years, children often mis-segment the acoustic signal, such that ‘words’ for an individual child may be idiosyncratic: e.g., *gu-daw* may become the label for dogs, if the infant consistently hears ‘good dog’ when the family dog is present. Once the infant realizes that objects have labels and actions have names, still other learning devices are recruited. The child will start pointing to or presenting objects to caregivers as a way of requesting the label. Critically important is the idea that these early words are holistically represented for the child, in production as well as in perception. Different authors have used varying terms to convey this idea. For example, Vihman and Velleman [22] called them ‘word recipes’ and Menn [23] called them ‘articulatory routines.’ But regardless of what term is used, the general idea is that words lack phonetic, articulatory, and acoustic detail in the child’s early representations. In fact, Charles-Luce and Luce [24] demonstrated that early lexicons are shaped by the child’s goal of keeping words in the lexicon maximally different in acoustic terms. These representations fit within the constraints imposed by auditory functions in these early years because sensitivity to detailed acoustic structure is not required. Over the course of much of childhood, these holistic representations are refined into segmental (i.e., phonological) structure, a process termed *lexical restructuring* [25–30].

Children are undoubtedly refining their lexical representations through the early years, but towards the end of preschool the process accelerates. Children begin to recognize word-internal structure more clearly and become facile at isolating and manipulating these briefer (phonological) units such as syllables and phonemes [17,25,27,31–34]. This change in sensitivity to linguistic structure coincides with burgeoning perceptual skills, including a shift in attention to the acoustic cues that are most informative regarding phonemic structure in the child’s native language [20,35]. The premise serving as the basis of the analyses reported here was that this period of relatively rapid phonological discovery at the end of the preschool years requires that auditory development is progressing in typical fashion and is sufficient by this age to support the discovery of phonological representations. It is hypothesized that children’s sensitivity to phonological structure during this critically important period will be strongly related to their sensitivity to acoustic structure, especially temporal and spectral modulation. It is further hypothesized that lexical and syntactic knowledge will not be as strongly related to sensitivity to acoustic structure, because words can be and in fact are represented more holistically for young children. Later in childhood, items entering the lexicon, such as those used in school, will come to rely more strongly on refined phonological representations as they become more complex [36,37], but for young children, they are underspecified compared to words in adults’ lexicons. Knowledge of syntactic rules – at least those involving word order – can be acquired by learning how to arrange the holistic lexical elements of early childhood. To test these hypotheses, children’s sensitivity to both temporal and spectral modulation was measured, and measures were collected of lexical and syntactic knowledge, as well as of phonological sensitivity.

### Preschool critical periods

Many developmental phenomena do not show linear growth. Instead, periods of rapid emergence exist, with relatively stable performance before and after those periods. During these periods of rapid emergence there is typically wide variability among children as some develop the skill sooner within that window than others. If subjects are recruited at ages younger than that period of rapid emergence, little variability will be found across subjects because even those children developing typically will not have started to acquire the skill. And at later ages it might be that all children, even those late in developing the skill under study, will have achieved mastery. Thus, it is essential to select the age range at which maximum variability is expected for the skill being examined. For both the development of sensitivity to temporal and spectral modulation and acquisition of phonological sensitivity, that age is best defined as the end of preschool, as children are entering primary school and beginning to learn to read.

Results of the study cited earlier to describe temporal modulation transfer functions [8] can also be used to illustrate the concept of critical periods and to support the selection of the age range used in this study. Nittrouer and Lowenstein [8] investigated children’s sensitivity to temporal modulation, along with phonological sensitivity; 117 children between the ages of 5 and 10 years participated. The phonological sensitivity task in that experiment was a final consonant choice (FCC) task, in which the child was presented with a word that needed to be repeated. Three words were then presented and the child needed to identify the one that ended in the same sound.

**Fig 5:**
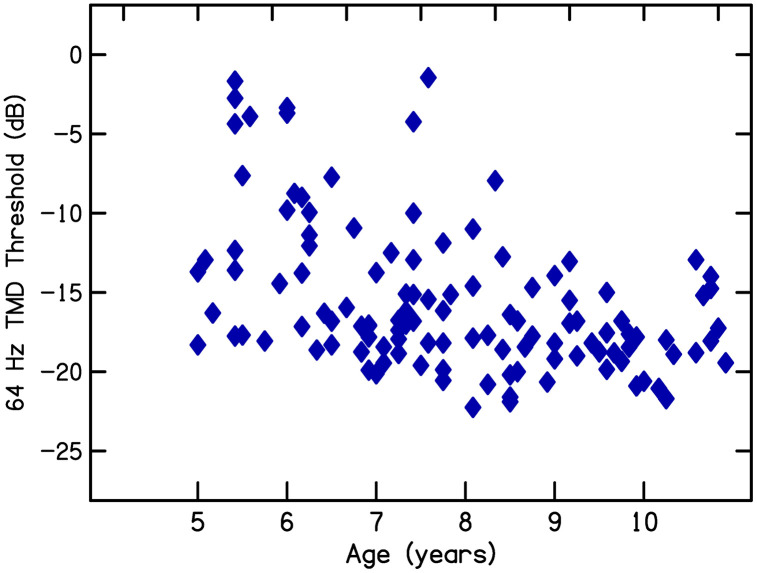
Scatter plot of detection thresholds for temporal modulation at a 64-Hz modulation rate as a function of listener age (data from [8]).

**Fig 6:**
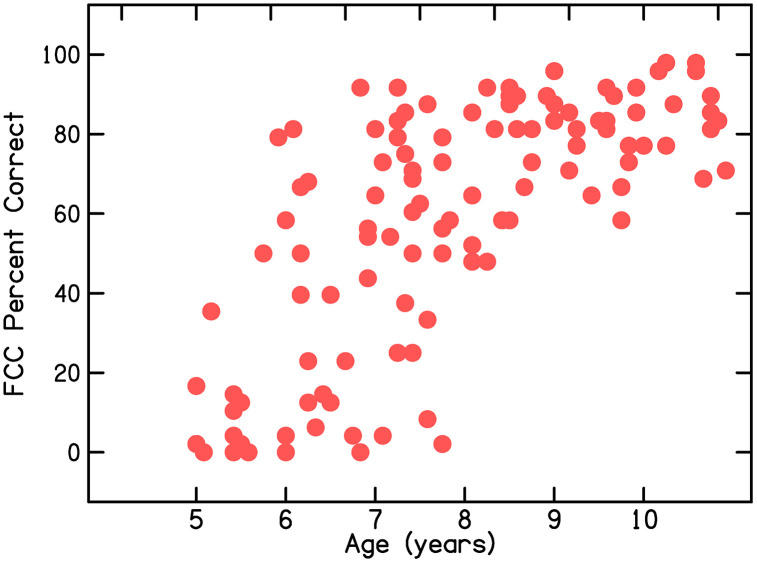
Scatter plot of scores on the final consonant choice (FCC) task as a function of listener age (data from [8]).

Fig 5 displays the relationship between age and temporal modulation detection thresholds for signals with a 64-Hz modulation rate. Fig 6 displays the relationship between age and scores on the FCC task. Developmental changes are apparent and significant correlations with age are found for both measures: for temporal modulation detection, *r*(117) = −.449, *p* < .001, and for FCC, *r*(117) =.695, *p* < .001. A critical characteristic seen in these figures, however, is the developmental reduction in variability for both measures. This reduced variability is the characteristic that most strongly supports the selection of the age range of 5–6 years for the current study, because it is the age at which there is most variability among children for these auditory and language phenomena; in essence, it is the sensitive, or critical, period for learning these skills. The central question of this investigation was whether development of language knowledge in this age range, especially phonological sensitivity, is related to development of these auditory functions.

### Maximizing variability

In the current study, variability in both language and auditory development was sought through subject selection. Two conditions were targeted that are known to be associated with language development: socioeconomic status and gestational age at birth. For each condition, there was reason to suspect that the development of the central auditory pathways (those above the level of the auditory nerve) is affected, and that was the hypothesis explored in this study. Each condition was treated as continuous in nature, rather than as discrete categories, on the assumption that effects should be continuous in magnitude.

Socioeconomic status is well recognized as a factor associated with language attainment. This construct is typically defined as a composite value of the educational level and occupational prestige of the primary income earner in the home, although maternal education sometimes serves as the independent variable. Children living in abject poverty have reliably been found to perform more poorly on measures of language ability than their middle-class peers [38–43], but evidence shows that socioeconomic status influences child-language outcomes even at the mid- to high-end of the socioeconomic spectrum. Hoff [44] reported poorer language outcomes for children whose parents had no education beyond high school than for children whose parents had graduated from university. And although poverty has been found to negatively impact other functions, such as attention and inhibition, it takes its heaviest toll on language [45]. Traditionally, this delay has been attributed to social determinants, mostly deficiencies in the quantity and quality of language models available in the child’s environment [46–49]. Correlations between amount of language input and child language performance, however, usually fail to explain more than 30 percent of the variance in language abilities (e.g., [49,50]), leaving open the question of what other poverty-related factors explain the poor language outcomes. We suggest that delayed development of the central auditory pathways may explain some of that variance, a suggestion motivated by findings showing that poverty is associated with changes in brain structure, including reduced gray matter in the frontal and temporal cortices, as well as in the hippocampus [51, 52]. Socioeconomic status has also been associated with neural activity at the cortical level, such that higher socioeconomic status promotes stronger activity in language-relevant regions [53]. Accordingly, it was reasonable to propose that socioeconomic status might specifically affect the development of the central auditory pathways, and to examine that proposal with behavioral measures of function in those pathways. Of special interest is evidence that poverty specifically disrupts development of specialization of the inferior frontal gyrus (Broca’s area) [54]; this finding provides further support that socioeconomic status may be especially related to development of phonological sensitivity.

Children born prematurely demonstrate both poorer language abilities and auditory functioning than children born full term. For example, children born at all levels of premature status perform more poorly on language measures than their full-term peers [55–57], and these deficits are observed even when subjects born prematurely are matched to full-term subjects on other factors, including maternal education and IQ [58]. In fact, there is evidence that these language difficulties increase as children mature [59]. Where auditory functions are concerned, children born prematurely display “unstable” auditory performance through at least the first year of life, meaning auditory-evoked potentials vary in an unpredictable manner [60]. As late as 8–10 years of age, it has been observed that premature children demonstrate poor temporal ordering and gap-in-noise detection [61]. These auditory deficits are present even when auditory thresholds are within the normal range [62], indicating that they are most likely due to impaired central auditory functions. The altered acoustic environment of the neonatal intensive care unit, compared to the intrauterine acoustic environment, is typically considered the likely cause of the language and auditory deficits exhibited by children born prematurely [63,64]. However, results from animal models of premature birth, where other variables are kept constant, show that the simple act of being removed from the womb early results in neurological deficits [65].

### Summary

Language is a uniquely human trait, but it is not always acquired without challenges. Delays or deficits in language and literacy development are the most common problems children face – more common than autism or sensorineural hearing loss [66]. If not effectively treated, language deficits in childhood can become lifelong problems, diminishing almost every aspect of an individual’s life. The investigation described here was undertaken to explore the broad proposal that delays in the development of the central auditory pathways can restrict language development, leading to deficits in language abilities. To explore this proposal, two auditory functions were examined as potential markers of that auditory development: sensitivity to temporal and spectral modulation. The specific proposal offered here is that sensitivity to acoustic structure in the speech signal is more important for the development of phonological representations than for lexicosyntactic abilities. To examine this proposal, measures of lexical, syntactic, and phonological abilities were obtained across subjects. Variability in both language and auditory performance was maximized by recruiting subjects across a set of conditions predicted to impose variability in developmental timing for both kinds of phenomena.

Two specific hypotheses were tested in this work: (#1) The timely development of auditory functions more strongly affects later-emerging sensitivity to phonological structure than earlier-emerging lexicosyntactic abilities; and (#2) Two conditions that have been shown to negatively influence language development actually exert that influence – at least in part – by interfering with the development of auditory functions, namely, socioeconomic status and gestational age at birth.

## Methods and materials

All methods used in this study were approved by Institutional Review Board 1 (IRB1) of the University of Florida under protocol IRB202301499 prior to the start of data collection. Approval was obtained on November 6, 2023, and recruitment began on November 7, 2023. Recruitment is ongoing.

### Participants

Subjects were recruited for this study through several methods. Both paper and digital flyers were distributed to schools, community centers, libraries, and medical clinics. Distribution efforts were strongly focused in low-income neighborhoods. Additionally, electronic records of the university medical system were searched for diagnostic codes associated with premature birth for patients whose parents had agreed at a previous clinic visit to be contacted about potential research studies. Parents of the patients identified through those efforts were contacted about possible participation through letters and emails. To qualify, children needed to be between 5;0 and 7;0 years of age. They had to have normal hearing and no frank disabilities that are known to affect language development, other than possibly low socioeconomic status or premature birth. They could not have experienced an intracranial hemorrhage at any time, and could not have had more than five diagnoses of otitis media of any sort made by an otolaryngologist before the age of 3 years. From this process, parents of 114 children contacted us about possible participation. Four of these children came to the laboratory but were dismissed before being consented: one because of active otitis media on test day and three because they presented with autism. Thus, 110 children were consented. One of these children was dismissed partway through testing due to a conduct disorder and another because it became clear partway through testing there was a significant developmental delay. One child’s data were more than 3 SDs from the mean of the other children on all tasks, so that child’s data were not included. Review of medical records for three children revealed periods of raised auditory thresholds earlier in life, so their data were not included in these analyses. In total, data from 104 children were included.

Fifty-one percent (53) of these subjects were male and 49 percent (51) were female. Twenty-eight percent (29) of these subjects were identified by their mothers as Black, 52 percent (54) as White, 3 percent (3) as Asian, and 17 percent (18) as Multiracial. All children identified as Multiracial had one parent who identified as Black and one parent who identified as White. All children included in the study passed a hearing screening consisting of the octave frequencies between 0.5 and 4.0 kHz at 20 dB hearing level for both ears, as well as an otoacoustic emissions screening. None of the children had flat tympanograms at the time of testing. All children were administered the figure-ground subtest of the Leiter International Performance Scale – Revised [67] and had scaled scores above 7 (i.e., better than −1 SD). Forty-nine percent (51) of the children had public health insurance (i.e., Medicaid).

Table 1 displays means, medians, and SDs for several independent variables. Socioeconomic status was a two-factor index, computed for each child using a formula described previously by Nittrouer and Burton [68]. This index was based on that of Hollingshead [69] and involves scaling from 1 to 8 both the educational attainment and occupational status of the parent in the household with the highest income; these values are multiplied together to produce an index between 1 and 64 (low to high). The index we used differs from that of Hollingshead primarily in that occupations were updated to incorporate technical occupations that did not exist in the 1950s and to remove occupations that no longer exist. The values used to compute socioeconomic status were obtained from the subject’s mother. In general, scores below 15 indicate abject poverty where the primary income earner has at most a high school diploma and is unemployed or does not have stable employment. Thirty-six percent (37) of the children in this study were in this category. Scores between 16 and 29 generally indicate that the highest income earner has a high school diploma or possibly an associate’s degree and is working in a service industry or in a retail sales position; they may or may not have stable employment. Nineteen percent (20) of the children in this study were in this category. Scores between 30 and 42 generally indicate that the primary income earner has a four-year university degree and a stable career at a level that is considered middle class. Twenty-eight percent (29) of the children in this study were in this category. Scores above 42 indicate that the primary income earner has at least a master’s degree or more often a higher degree and a more prestigious job, such as a university professor, medical doctor, or attorney. Seventeen percent (18) of the children in this study were in this category.

**Table 1:** Means, medians, and SDs for independent variables. N = 104.

*Independent variables*	Mean	Median	SD
Age (months)	71.4	70.5	7.2
Socioeconomic status	25.8	25.0	16.1
Maternal education (years)	13.0	13.0	1.5
Gestational age at birth (weeks)	37.6	39.0	3.5
Birthweight (grams)	3054	3189	860
Time in neonatal intensive care (days)	7.6	0.0	24.8
Figure-ground scaled score	9.9	10.0	3.1

Maternal education was obtained from mothers, as well. Although the education values used in the computation of socioeconomic status were for the highest income earner, which could mean the child’s father, maternal education correlated highly with socioeconomic status, *r*(104) =.822, *p* < .001. Values for gestational age at birth, birth weight, and time in the neonatal intensive care unit were obtained from the subject’s mother, and were confirmed from the children’s medical records. Both birth weight and time in the neonatal intensive care unit correlated highly with gestational age at birth: birthweight, *r*(104) =.823, *p* < .001, and time in the neonatal intensive care unit, *r*(104) = −.779, *p* < .001.

### Equipment

Hearing screenings were performed with a Grason-Stadler Gsi 61 audiometer and TDH-39 headphones. These hearing screenings, as well as testing for the modulation detection tasks, auditory comprehension of language, and the phonological sensitivity tasks took place in a soundproof booth. Figure-ground and vocabulary testing took place in a quiet room. Tympanometry was performed with an Interacoustics Titan. Otoacoustic emissions were obtained with a Grason-Stadler Corti. Stimuli for both the modulation detection and phonological sensitivity tasks were presented through a computer, with a Creative Labs Soundblaster soundcard, a Samson C-Que 8 amplifier, and AKG-K141 headphones. Children’s responses for the figure-ground, vocabulary, auditory comprehension of language, and phonological tasks were video recorded using a SONY HDR-XR550V video recorder so that responses could be checked later by independent staff members.

### Stimuli and task-specific procedures

#### Audiometric Testing.

Children had their hearing screened using behavioral methods at the octave frequencies between 0.5 and 4.0 kHz at 20 dB hearing level for each ear. In addition, distortion-product otoacoustic emissions were obtained for 2.0, 3.0, 4.0, and 5.0 kHz and tympanograms were obtained. Each child needed to pass the behavioral screening and obtain a pass (rather than a refer) with otoacoustic emissions. A Type B tympanogram was an exclusionary criterion.

#### Modulation detection.

Stimuli for measuring temporal modulation detection were generated in Matlab and consisted of broadband noise (0.05 to 8.0 kHz). Standard stimuli had flat envelopes. Target stimuli were sinusoidally amplitude modulated in the time domain at one of two rates: 8 Hz or 64 Hz. Both these rates are below the 3-dB cut-off found for listeners in [8], as shown in Fig 2. All stimuli had 20-ms cosine-squared onset and offset ramps. RMS amplitude was equalized across stimuli and duration of all stimuli was 500 ms. Modulation depth (m) varied between 0 and 1, and is described in dB derived from 20*log(m). Initial depth was 0 dB (maximum depth), with a step size of 4 dB for the first 2 reversals, and 1 dB for the next 8 reversals. These stimuli were presented at 68 dB sound pressure level. Detection thresholds were defined as the means of the last eight reversals. More negative thresholds represented better temporal modulation detection.

Stimuli for measuring spectral modulation detection were also generated in Matlab. Each stimulus consisted of 800 sinewave components of equal amplitude, logarithmically spaced between 0.1 and 5.0 kHz. Starting phase of each component was randomly selected on each trial. These stimuli had flat spectra and were used as the standards. Target stimuli were created by applying a sinusoidal envelope with a modulation rate of 0.5 cpo to the spectrum. Starting phase of that modulated envelope changed randomly across trials. The overall envelope was further shaped in two ways. First, amplitude of the lowest frequency components increased gradually, and amplitude of the highest frequency components decreased gradually. Second, a speech-shaped envelope [70] was applied. These measures ensured that there were no large amplitude discontinuities at stimulus edges. RMS amplitude was equalized across stimuli, and duration of all stimuli was 500 ms. Modulation amplitude for these stimuli is given in dB change from peak to trough. In this task, the original depth of modulation was 30 dB peak to trough and step size was 4 dB for the first two reversals. Step size then changed to 2 dB where it remained for eight more reversals. These stimuli were presented at 68 dB sound pressure level, roving by +/-3 dB. The detection threshold was defined as the mean of the last eight reversals.

A three-alternative, forced-choice procedure created in Matlab was used in a two-down, one-up adaptive paradigm to obtain the thresholds for 70.7% correct detection for both temporal and spectral modulation. Two runs of each of the three stimulus sets were presented, and the mean thresholds across the two runs was used as measures in further analysis.

In this task, the child saw a display on the computer monitor of either three cartoon robots or three cats in a horizontal array. The child was told that one of the characters made a different sound from the others. As each of the three stimuli was played, each of the characters pulsed, in turn from left to right. The child needed to identify the one that was different.

Prior to testing, the child was given practice identifying stimuli at the greatest modulation depth. The child had to identify the modulated stimulus correctly on nine out of ten consecutive trials without prompting to move to testing. They had a maximum of 30 stimuli in which to meet this criterion. Six children failed to meet this practice criterion for 8-Hz temporal modulation, 16 failed to meet the criterion for 64-Hz temporal modulation, and 18 children failed to meet the criterion for spectral modulation. If a child failed to meet the practice criterion, maximum depth of modulation was used as their threshold for that run on the premise that it was the best estimate possible. Because all children who failed to pass training on one of these runs did pass on at least one of the other tasks, it was concluded that these children could perform a three-interval forced choice procedure.

Several steps were taken to ensure that children were attending to the task throughout testing. First, ‘easy’ trials consisting of target stimuli with the deepest modulation were presented every four to six trials. Children were required to identify these stimuli correctly (i.e., no more than two incorrect responses per run) for their data for that run to be included in analysis, a criterion they all met. In general, children did not make errors on these easy trials: mean numbers of errors were less than 1.0 for all three modulation conditions. The possibility was considered, however, that problems in attention might arise only near threshold. To assess that possibility, two additional measures were obtained across the last eight reversals: the SD of those reversals and the mean length of excursion (MLE), which was the mean distance between each consecutive pair of reversals for the last eight reversals.

**Fig 7:**
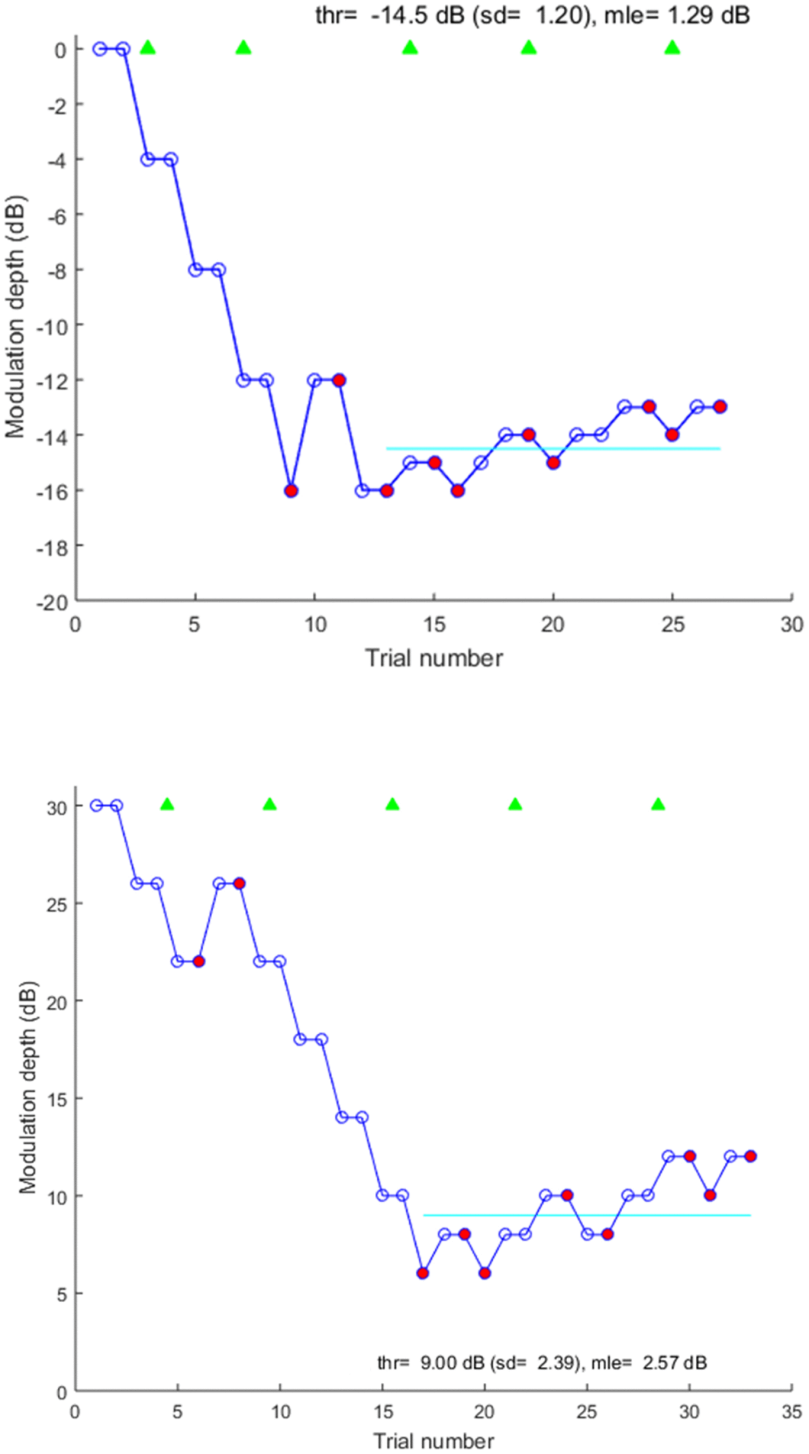
Adaptive tracks from one child for temporal modulation detection at 8 Hz (top) and spectral modulation (bottom). Red circles are reversals and green triangles are responses to easy trials.

Fig 7 shows adaptive tracks from one child. The top track is for temporal modulation at the 8-Hz modulation rate and the bottom figure is for spectral modulation. The red circles show the reversals. The green triangles indicate that responses to all easy trials were correct.

#### Vocabulary.

Vocabulary knowledge was measured with the Expressive One-Word Picture Vocabulary Test – 4^th^ Edition [71]. In this task, a series of pictures are shown and the child is asked to label each one. The vocabulary items become less common as the test proceeds. Testing stopped after six consecutive errors. All testing was recorded and reviewed later to ensure correct scoring. Standard scores are given in Table 2 to provide a picture of the sample, but raw scores were used in analyses.

**Table 2:** Means, medians, and SDs for dependent measures for the current study. N = 104.

*Dependent measures*	Mean	Median	SD
Vocabulary (standard score)	101.7	100.5	14.1
Auditory comprehension (standard score)	96.4	96.5	13.8
Initial consonant choice (percent correct)	44.0	43.8	33.8
Rhyme oddity (percent correct)	48.2	50.0	31.0
Temporal modulation 8 Hz (dB)	−12.1	−13.2	6.2
Temporal modulation 64 Hz (dB)	−10.6	−11.9	5.9
Spectral modulation (dB)	19.3	19.4	8.3

#### Auditory comprehension of language.

Children’s abilities to comprehend spoken language were assessed using the auditory comprehension subtest of the Preschool Language Scales – 5 [72]. This task requires the child to demonstrate an understanding of spoken language by performing specific commands given by the examiner. Testing stopped after six consecutive errors. All testing was recorded and reviewed later to ensure correct scoring. Standard scores are shown in Table 2, but raw scores were used in analyses.

#### Phonological sensitivity.

Children were administered two tasks assessing phonological sensitivity. This sensitivity emerges at different developmental stages according to level of structure [73,74], with sensitivity to syllable units appearing first, then rimes, followed by syllable onsets, and finally syllable offsets. For 5- and 6-year-olds, sensitivity to rimes and syllable onsets is most strongly emergent, so tasks were selected to assess these levels of sensitivity. A rhyme oddity task was used to assess the first of these, children’s sensitivity to rime structure. In this task, the child saw three cartoon faces on the computer monitor and heard three words. The faces pulsed in order as each word was presented, from left to right. The child’s task was to select the word that did not rhyme. Six practice items were provided. The experimenter initially coached the child, if needed. If the child could not respond correctly to any of the practice trials without coaching the first time they were presented, these practice trials were repeated. Testing then commenced. There were 36 items in this test, and testing was discontinued if the child responded incorrectly to six consecutive trials. The percent correct score was used as the dependent measure.

The other task of phonological sensitivity assessed children’s abilities to match sounds at the beginnings of words. In this task, the initial consonant choice (ICC) task, subjects were first presented with a target word, which they had to repeat. Auditory presentation of this target word was accompanied by the presentation of a cartoon face in the middle of the computer monitor. Next the child saw three faces and heard three word choices. The faces pulsed one at a time from left to right as each word was presented. The child had to select the one that started with the same ‘sound’ (i.e., consonant) as the target word. Before testing, six practice trials were presented. Then testing commenced. Testing was discontinued if the child responded incorrectly to six consecutive trials. There were 48 items. The percent correct score was used as the dependent measure.

Stimuli in both phonological sensitivity tasks were presented at 68 dB sound pressure level. All testing was recorded and reviewed later to ensure correct scoring.

### General procedures

Children came to the laboratory with their mothers. All testing was completed in one day during four sessions with breaks between each session. Lunch was provided between the second and third sessions. Consent was obtained before testing started. The consent form was sent to parents at the time they scheduled participation, so they had time to read it before coming to the laboratory. When they arrived, the mother and child were given a ‘tour’ of the laboratory, showing them where they would be participating; the tasks were explained. They were given the opportunity to ask questions. Parents signed the consent form on behalf of their children, and children gave verbal assent, witnessed by a staff member.

In the first session, audiologic testing was completed, along with the figure-ground and the vocabulary tasks. In the second session the three modulation sensitivity measures were administered, along with the ICC task. The third session consisted of the auditory comprehension of language task and the rhyme oddity task. The last session consisted of the three modulation sensitivity measures again. Parents were compensated $100 for their participation in the study. Children got a small toy at the end of each session and a T shirt at the end of the day.

## Results

SPSS was primarily used for statistical analyses. Data screening demonstrated that the four language measures and the three measures of auditory function were normally distributed.

### Description of results

Table 2 displays means, medians, and SDs for the two lexicosyntactic measures, the two phonological sensitivity measures, and the three measures of modulation detection. Four of these scores can be compared to those from the 5- and 6-year-olds whose data are included in Figs 5 and 6: vocabulary, ICC, temporal modulation detection at 64 Hz, and spectral modulation detection. The same vocabulary test was administered in that study as was administered in the current study, and ICC was administered, as well as FCC in that previous study. Means (and SDs) for children from [8] are shown in Table 3; these scores are only for the children with no histories of chronic otitis media. In comparing scores in Table 2 for children in the current study with scores in Table 3 for children in that earlier study it can be seen that, overall, children in the current study performed more poorly. This difference is likely because scores for the children in the current study include children varying in socioeconomic status and gestational age at birth; these children from that previous study had a mean socioeconomic score of 34.3 (median = 35) and none were born prematurely. Nonetheless, the scores in Table 3 provide benchmarks of best performance for children in this age range, meaning for middle-class children who were not born prematurely and did not have any other risk factors for language delay.

**Table 3:** Means, medians, and SDs for dependent measures from [8] that the current study shares with that study for 5- to 6-year-olds. N = 22.

*Dependent measures*	Mean	Median	SD
Nittrouer & Lowenstein [8]			
Vocabulary standard score	115.1	116.0	11.5
Initial consonant choice	77.1	77.1	16.5
Temporal modulation 64 Hz	−14.5	−16.9	4.8

The measures collected to examine children’s response reliability were examined, as well. These were the measures of SD and MLE for the last eight reversals. Table 4 shows means, medians, and SDs for these values in each of the three conditions. Clearly, these values are not much larger than the 1-dB step size used in measuring sensitivity to temporal modulation or the 2-dB step size used in measuring spectral modulation. In sum, these 5- and 6-year-olds were able to maintain attention to these tasks.

**Table 4:** Means, medians, and SDs for the SDs and mean lengths of excursion (MLEs) in dB for the three measures of modulation detection. N = 104.

*Modulation detection task*	SD			MLE		
	Mn	Mdn	SD	Mn	Mdn	SD
Temporal modulation 8 Hz	2.3	2.1	1.2	2.2	2.1	1.2
Temporal modulation 64 Hz	2.0	1.9	1.1	2.1	2.0	1.0
Spectral modulation	2.4	2.6	1.5	3.0	3.5	1.7

Note: Mn = mean; Mdn = median

### Tests of Hypothesis #1

This hypothesis was that the development of the suprathreshold auditory functions examined here more strongly affects acquisition of phonological sensitivity than lexicosyntactic knowledge. As prelude to testing this hypothesis, Pearson correlation coefficients were computed between each of the three modulation detection thresholds to see if all these measures were assessing the same phenomenon, presumably the development of the central auditory pathways. For the two measures of temporal modulation detection, a high correlation was found, *r*(104) =.756, *p* < .001. Where spectral modulation detection was concerned, high correlations were also found: for 8-Hz temporal modulation, *r*(104) =.658, *p* < .001; and for 64-Hz temporal modulation *r*(104) =.647, *p* < .001. Thus, it was concluded that all three measures of modulation detection largely evaluate the same phenomenon; nonetheless, the way that these specific aspects of auditory function support language acquisition may differ.

Moving to actual tests of the hypothesis, Table 5 displays Pearson correlation coefficients for each of the three measures of modulation detection and each of the lexicosyntactic and phonological sensitivity measures. All these correlation coefficients are significant, and medium to large in effect size. There is also a clear pattern across language tasks: Each modulation detection threshold had the smallest correlation coefficient for vocabulary, and the highest for one or the other phonological sensitivity task: ICC or rhyme oddity. Correlation coefficients between the modulation detection tasks and the auditory comprehension task were intermediate with those for vocabulary and the two phonological sensitivity measures, except where auditory comprehension is concerned: that correlation with 64-Hz temporal modulation is slightly higher than the correlation between ICC and 64-Hz modulation.

**Table 5:** Correlation coefficients between each of the three measures of modulation detection and each of the lexicosyntactic and phonological sensitivity measures. N = 104.

*Modulation detection task*	Vocabulary	Auditory Comprehension	Initial Consonant Choice	Rhyme Oddity
Temporal modulation 8 Hz	−.323***	−.484***	−.520***	−.564***
Temporal modulation 64 Hz	−.377***	−.464***	−.447***	−.486***
Spectral modulation	−.431***	−.501***	−.578***	−.543***

NOTE: df = 104 for all; ***, *p* < .001

Although informative, these individual correlation coefficients do not account for the likelihood that the three measures of auditory function had overlapping effects on the language measures. Therefore, stepwise regression was conducted to examine the unique contributions of each auditory function to each of the lexicosyntactic and phonological sensitivity measures. These results, shown in Table 6, reveal that spectral modulation detection explained the most variability in three of the four language measures; temporal modulation detection at 8 Hz explained the most variability in the fourth, rhyme oddity. For all language measures, temporal modulation detection at 64 Hz explained no additional variability once spectral modulation detection thresholds and thresholds for temporal modulation detection at 8 Hz were incorporated. Finally, reflecting the pattern of results for the correlation coefficients shown in Table 5, it can be seen in Table 6 that the auditory functions explained the least amount of variability for vocabulary and the most for the two measures of phonological sensitivity; the amount of variability in auditory comprehension explained by these auditory functions was intermediate.

**Table 6:** Stepwise regression outcomes for each of the lexicosyntactic and phonological sensitivity measures. Predictor variables were detection thresholds for spectral modulation, 8-Hz temporal modulation, and 64-Hz temporal modulation. N = 104.

*Dependent measures*			
Vocabulary			
	Significant Predictor Variables	Standardized Coefficients	R^2^
Step #1	Spectral modulation	*β* = -.431, *p* <.001	.186
Auditory Comprehension			
	Significant Predictor Variables	Standardized Coefficients	R^2^
Step #1	Spectral modulation	*β* = -.501, *p* <.001	.251
Step #2	Spectral modulation Temporal modulation 8 Hz	*β* = -.323, *p* =.005 *β* = -.271, *p* =.016	.293
Initial Consonant Choice			
	Significant Predictor Variables	Standardized Coefficients	R^2^
Step #1	Spectral modulation	*β* = -.578, *p* <.001	.335
Step #2	Spectral modulation Temporal modulation 8 Hz	*β* = -.416, *p* <.001 *β* = -.247, *p* =.021	.369
Rhyme Oddity			
	Significant Predictor Variables	Standardized Coefficients	R^2^
Step #1	Temporal modulation 8 Hz	*β* = -.564, *p* <.001	.318
Step #2	Temporal modulation 8 Hz Spectral modulation	*β* = -.363, *p* <.001 *β* = -.304, *p* =.005	.370

The results described above demonstrate that these suprathreshold auditory functions explain a moderate amount of variability in both the lexicosyntactic and phonological sensitivity measures. The crux of Hypothesis #1, however, was that these auditory measures would explain more variability in phonological sensitivity than in lexicosyntactic knowledge. From Tables 5 and 6 it appears the hypothesis was supported. In Table 5, it is found that thresholds for all three measures of modulation detection were more strongly correlated with ICC and rhyme oddity than with vocabulary or auditory comprehension, except for 64-Hz temporal modulation, which was more strongly correlated with auditory comprehension than with ICC. In Table 6, R^2^ was greater for ICC and rhyme oddity than for vocabulary or auditory comprehension, indicating that these auditory functions explained more variability in the phonological sensitivity measures than in the lexicosyntactic measures. Nonetheless, it was necessary to examine whether these differences in correlation coefficients between the kinds of language skills were significant in order to support fully the conclusion that the auditory measures were more strongly related to phonological sensitivity than to lexicosyntactic knowledge. To do so, we considered each measure of modulation detection separately, comparing its relationship with each lexicosyntactic measure to its relationship with each phonological sensitivity measure. Using R software, test statistics were calculated as the difference between the two correlation coefficients divided by the corresponding standard error. Standard errors and *p*-values were calculated using nonparametric bootstrapping procedures. Derived test statistics are shown in Table 7. Using an alpha of.05, three of these comparisons are significant, all involving the vocabulary measure: the correlation of spectral modulation and ICC is greater than the correlation of spectral modulation and vocabulary, plus the two correlations involving 8-Hz temporal modulation are greater for phonological sensitivity than for vocabulary knowledge. Although the test statistic for spectral modulation and vocabulary versus rhyme oddity does not reach the.05 probability level, it is close and less than.10. Thus, the pattern of results that emerges from this set of test statistics matches what has been seen in Tables 5 and 6: the relationships between the auditory functions and vocabulary knowledge are the weakest and most likely to be significantly different from what is found for the auditory functions and phonological sensitivity. Although correlation coefficients were smaller, in general, for the auditory functions and auditory comprehension, they were not statistically different from those found for auditory functions and phonological sensitivity.

**Table 7:** Test statistics, and probabilities, comparing the magnitude of the correlation coefficient for each measure of modulation detection and each of a lexicosyntactic measure versus a phonological sensitivity measure. N = 104.

*Modulation detection task*	Vocabulary versus		Auditory comprehension versus	
	Initial consonant choice	Rhyme oddity	Initial consonant choice	Rhyme oddity
Spectral modulation	1.71, 0.043	1.29, 0.096	1.29, 0.105	0.60, 0.274
Temporal modulation 8 Hz	2.74, 0.011	2.57, 0.004	0.53, 0.291	0.98, 0.154
Temporal modulation 64 Hz	0.76, 0.219	1.11, 0.128	−0.25, 0.492	0.27, 0.386

### Tests of Hypothesis #2

The second hypothesis tested in this study was that factors previously shown to negatively impact language acquisition would be found to take their toll in part or wholly through their effects on the development of auditory functions. To test this hypothesis, we first correlated the independent variables of socioeconomic status and gestational age at birth with both our language and auditory measures. Table 8 displays Pearson correlation coefficients between each of the two independent variables and each of the lexicosyntactic and phonological sensitivity measures, as well as the measures of auditory function. Both socioeconomic status and gestational age at birth were significantly correlated with all four language measures, as well as with the measures of auditory function.

**Table 8:** Correlation coefficients between each of the two independent variables and each of the lexicosyntactic and phonological sensitivity measures. N = 104.

*Independent variable*	Vocabulary	Auditory Comprehension	ICC	Rhyme Oddity	Temp 8	Temp 64	Spectral
Socioeconomic status	.429***	.356***	.299**	.413***	−.256**	−.289**	−.235*
Gestational age (weeks)	.255**	.323***	.316**	.334***	−.316**	−.268**	−.368***

NOTE: df = 104 for all; *, *p* < .05; **, *p* < .01; ***, *p* < .001

Socioeconomic status is a major source of variability in language acquisition, and the mechanism typically invoked to explain this effect involves language models in the environment, which are lacking in both quantity and quality for children in poverty (e.g., [46]). We hypothesized that socioeconomic-related effects on the development of the central nervous system, including the auditory pathways, also contribute to the observed relationship between socioeconomic status and language acquisition. Comparing the coefficients displayed in Table 8 to those displayed in Table 5 provides some support for that hypothesis. Table 5 shows that auditory functions are strongly associated with language measures. Table 8 shows that socioeconomic status is significantly correlated with both auditory functions and language measures, although the strength of the association with language measures is typically less than the association between auditory functions and language measures. The combination of these results could be explained by the fact that the effect of socioeconomic status on language measures is at least partly through an effect on auditory functions, which in turn affects language development. Testing this directly would require mediation analysis, which we could not do because it requires repeated data over time for a stable sample. Instead, we took an alternative approach, which was to do partial correlation analysis to see if the associations between socioeconomic status and language measures decreased once we accounted for auditory functions. Similarly, we performed partial correlation analysis to see if the associations between auditory functions and language development decreased once we accounted for socioeconomic status (so all the other factors that covary with it). If the correlations between socioeconomic status and language development were found to be much smaller after accounting for auditory functions, but the correlations between auditory functions and language development remained high, even after adjusting for socioeconomic status, this would provide evidence that the effects of socioeconomic status on language development are partly through an effect on auditory functions. Similarly, if socioeconomic status was found not to have a significant partial correlation with language development after adjusting for auditory functions, it would suggest that all of the effect of socioeconomic status on language development is through an effect on auditory functions.

The choice of which modulation detection threshold to include in each analysis was based on the stepwise regression results shown in Table 6: For vocabulary knowledge, only thresholds for spectral modulation detection were included in the analysis because it was the only auditory function found to account for any significant amount of variability in vocabulary scores. For the other measures, detection thresholds for both spectral modulation and temporal modulation at 8 Hz were included, because each of these measures of auditory function accounted for significant amounts of variability. Table 9 shows results of these partial correlation analyses and reveals that both socioeconomic status and the modulation detection thresholds explained significant amounts of variability in the lexicosyntactic and phonological sensitivity measures, after controlling for the other. Nonetheless, it is also apparent that the auditory functions explained more variability when socioeconomic status was controlled (bottom row) than vice versa (top row). Thus, within the limits of this analysis, support is garnered for the hypothesis that socioeconomic status impacts language acquisition in some part through its negative effects on the development of the central auditory pathways.

**Table 9:** Partial correlation coefficients for socioeconomic status and modulation detection. N = 104.

*Predictor variables*	Vocabulary	Auditory Comprehension		Initial Consonant Choice		Rhyme Oddity	
Controlling for modulation detection							
Socioeconomic status	.374***	.261**		.180		.324***	
Controlling for socioeconomic status							
Modulation detection	Spectral	Spect.	Temp. 8	Spect.	Temp. 8	Spect.	Temp. 8
	−.431***	−.460***	−.435***	−.548***	−.481***	−.504***	−.520***

NOTE: For vocabulary, *modulation detection* included spectral modulation only; df = 102. For the other three measures, *modulation detection* included both spectral modulation (Spect.) and temporal modulation at 8 Hz (Temp. 8); df = 102; **, *p* < .01; ***, *p* < .001

Gestational age at birth is another factor known to be associated with language development. As with socioeconomic status, we were interested in examining the extent to which gestational age at birth and modulation detection were independent sources of variability in the language measures. To examine this question, we conducted partial correlation analyses, alternately controlling for either gestational age at birth or the relevant modulation detection thresholds, as we did with socioeconomic status. These results are shown in Table 10. In this case, gestational age at birth did not account for a significant amount of variability, once auditory functioning was accounted for. Modulation detection thresholds continued to account for significant amounts of variability. Therefore, within the limits of this analysis, it can be concluded that the primary pathway of effect of premature birth on language development is through its effect on the development of the central auditory nervous system.

**Table 10:** Partial correlation coefficients for gestational age at birth and modulation detection. N = 104.

*Predictor variables*	Vocabulary	Auditory Comprehension		Initial Consonant Choice		Rhyme Oddity	
Controlling for modulation detection							
Gestational age	.115	.152		.115		.146	
Controlling for gestational age							
Modulation detection	Spectral	Spect.	Temp. 8	Spect.	Temp. 8	Spect.	Temp. 8
	−.375***	−.435***	−.425***	−.524***	−.467***	−.480***	−.512***

NOTE: For vocabulary, *modulation detection* included spectral modulation only; df = 102. For the other three measures, *modulation detection* included both spectral modulation (Spect.) and temporal modulation at 8 Hz (Temp. 8); df = 102; ***, *p* < .001

## Discussion

This study was undertaken to explore the general proposal that rate of development of the central auditory pathways exerts an influence on language development by supporting or constraining the emergence of the language skills most dependent on auditory functioning. To explore this proposal, measures of temporal and spectral modulation detection were obtained, along with measures of language abilities from children in an age range when development of both sorts of skills should be maximally variable (i.e., during a sensitive period). Two specific and testable hypotheses were posed: (#1) The timely development of auditory functions more strongly affects later-emerging sensitivity to phonological structure than lexicosyntactic abilities, which begin to emerge earlier; and (#2) Several conditions known to negatively influence language development actually exert that influence, at least partly, by interfering with the development of auditory functions; conditions considered were socioeconomic status and gestational age at birth.

The first hypothesis was partly supported. Correlation coefficients were slightly, but consistently, stronger between the measures of auditory function and measures of phonological sensitivity than between measures of auditory function and lexicosyntactic knowledge, especially vocabulary, although these differences were not necessarily significant. An important nuance of these outcomes is that the reason this first hypothesis was only partly supported was that the correlations between auditory functions and lexicosyntactic knowledge were stronger than might have been expected. In fact, spectral modulation detection explained as much or more variability in lexicosyntactic knowledge than the independent variables considered in this study that are generally thought to be strongly related to language development: socioeconomic status and gestational age at birth. And it is not the case that weaker relationships were observed in this study than in previous studies between these independent variables and scores on the lexicosyntactic tasks. Results for socioeconomic status best illustrate trends across studies. Correlation coefficients between socioeconomic status and language performance are typically around.3. For example, Fernald et al. [50] found a correlation coefficient of.34 between vocabulary and socioeconomic status, as indexed by the Hollingshead index [69]. Here we obtained a correlation coefficient of.429 between vocabulary and our measure of socioeconomic status, which derived from the Hollingshead index. Thus, the effects of socioeconomic status on lexicosyntactic knowledge in this study resembled – or were somewhat greater than – what has been previously reported. All the same, effects of spectral modulation detection on vocabulary in this study were equal to, or slightly higher than the effects of socioeconomic status. Where auditory comprehension is concerned, all three measures of auditory function explained more variability in scores on this measure than did socioeconomic status or gestational age at birth. And in this case, none of the comparisons of correlation coefficients between the auditory measures and auditory comprehension versus phonological awareness were significant, revealing that the measures of auditory function explained close to the same amount of variance in auditory comprehension of language as in phonological sensitivity; this differs from findings for vocabulary, where four of the six test statistics were significant, or close to significant. This difference in outcomes for vocabulary and auditory comprehension might be explained by the fact that the auditory comprehension subtest of the Preschool Language Scales has some items that either explicitly test phonological sensitivity or indirectly test that sensitivity through measures of comprehension of inflectional morphemes. As a consequence, some part of the score on this auditory comprehension task likely reflects the phonological sensitivity of the test taker. Regardless, even though the finding that the development of these auditory functions was related to lexicosyntactic acquisition to some extent may weaken support for the first hypothesis, it strengthens the overall proposal that timely development of the central auditory pathways is critical to language acquisition.

Moderate support is also provided for the second hypothesis, that conditions previously found to influence language development may exert that influence (at least partly) by interfering with the development of auditory functions. Where socioeconomic status is concerned, partial correlation analysis revealed that the effects on language skills of the auditory functions and other factors related to socioeconomic status were to some extent independent: Each explained some unique variability in the language scores, although auditory functions explained more variability. Correlation coefficients between gestational age at birth and the language measures – lexicosyntactic and phonological – were also statistically significant. In this case, partial correlation analyses revealed that those effects of gestational age on the language measures ceased to be significant when auditory functions were controlled. Thus, gestational age – so premature birth – appears to take its toll on language acquisition entirely through whatever effect it has on the development of the central auditory pathways.

### Limitations

The major limitations of the current study were that it was largely correlational in design, and not all independent variables that may influence language outcomes were measured. Where socioeconomic status is concerned, no measures of parental language input were obtained for these particular children. The assumption was largely made here that language input serves as a social determinant of language, and the auditory functions we examined are biological determinants. However, it is possible that variability in the language children hear affects development of central nervous system structures, including the auditory pathways [75]. Future investigations will need to refine our understanding of the directions of effect of the variables associated with socioeconomic status and gestational age at birth, of which there are many. In the meantime, this study contributes to our current understanding of language acquisition and the factors that can promote or constrain it by demonstrating that the timing of development of certain suprathreshold auditory functions influences the development of language skills, especially those related to phonological sensitivity.

### Implications

The outcomes of this study contribute to models of language development and delay in several ways. There is likely no issue more controversial in the field of communication sciences and disorders than whether disruptions in auditory function beyond peripheral sensitivity can explain developmental language delays and disorders. Numerous studies have explored this idea that auditory dysfunctions are responsible for delays in language and literacy acquisition (e.g., [76–79]). The choice of auditory function and the selection of the language or literacy measure to include in these studies has varied, often without a clear vision of how the chosen auditory function would be expected to support acquisition of the specific language or literacy phenomenon being measured. In designing this study, we started with the selection of the language measures – lexical, syntactic, and phonological – from the perspective that phonological sensitivity begins to emerge later than children begin learning words and how to order them in sentences. Then we worked backwards to consider the auditory functions that would be the most likely candidates to support that later-emerging language skill of phonological sensitivity. In this way we were able to design a precise test of whether specific auditory functions support a specific language skill. Moreover, we carefully selected an age range in which it appeared that development of the specific functions under study would be progressing at the most rapid rate – an apparent sensitive period for the development of these auditory functions and phonological sensitivity. This age range should, it was predicted, provide the greatest variability across children and allow the strongest test of whether the emergence of these auditory functions supports, and so potentially constrains, the acquisition of phonological sensitivity. Generally speaking, the outcomes of these analyses supported the hypotheses offered.

Implications of these findings, however, are not limited to the age range studied here or to the childhood conditions examined. The notion of sensitive periods in development is well accepted where language acquisition is concerned (e.g., [80,81]), although the idea that there would be only one sensitive period for this development is generally eschewed (e.g., [82,83]). It is more likely that different components of language functioning emerge in a relatively consistent order, with periods of rapid emergence for each of those components (e.g., [84,85]). The auditory functions and phonological sensitivity phenomena examined in this study appear to emerge in large part during the age range studied. Other skills at least start to develop at earlier ages, and still other skills will only start to develop at later ages. The children in this study who were poor at the tasks examined – so were delayed in the development of these auditory and language skills – might nonetheless acquire these particular skills, albeit at a slightly older age. The implications of this late development are currently uncertain, but it seems a reasonable prediction that there would be a domino effect in which these children will be delayed with each emerging auditory and language function. The question becomes one of whether there is an age at which language acquisition ends, regardless of whether or not a child has successfully navigated each of those sensitive periods. Moreover, delays in auditory and language development surely have negative consequences for academic pursuits. If a child does not have the language skills expected at each grade level, the child will not be able to meet expectations for that grade.

Implications of these findings also extend to children beyond those who may be living in poverty or who may have been born prematurely because these conditions can co-occur with other risk factors for language delay. For example, children with congenital hearing loss may be living in poverty or may have been born prematurely; in fact, premature birth is a risk factor for hearing loss [86]. Because these conditions of poverty and premature birth were found, through behavioral measures, to hinder the development of central auditory pathways, we can predict that children with hearing loss who also experience one of these conditions will have poorer outcomes than children with hearing loss otherwise have.

One of the most significant implications of the findings from this investigation should concern how we view the effects of poverty on language acquisition. Unquestionably the predominant model of how poverty affects language acquisition is one involving social determinants. According to this model, the language environments of children in poverty are less supportive of language acquisition than the environments of children not living in poverty. In particular, it has been documented that women (mothers) in poverty talk to their children differently than women not living in poverty, by using more directives with no expectation of responses from their children and fewer linguistic devices that support the continuation of interactions (e.g., [46–49,87]. This model, however, has always suffered from a lack of large effect sizes. Moreover, even when differences in language input styles are controlled, children in poverty still demonstrate poorer language, with a lot of variance left unexplained. These results prompted Perkins et al. [41] to ask “…if poverty has a unique effect on language development, what is the mechanism through which poverty, controlling for other factors, influences language?” (p. 16). The study reported here helps to answer that question: At least one mechanism through which poverty influences language is its effect on the development of the central auditory pathways.

Another, though not mutually exclusive, proposal for how poverty affects language acquisition is the idea that poverty imposes stress on all members of the family unit [88]. That stress, it is hypothesized, is what is responsible for disruption in the development of the central nervous system [83,84]. Regardless of the precipitating factor, however, evidence clearly reveals poverty-related disruptions in central nervous system development [51,52,89]. In light of these demonstrated relationships between poverty and structure of the central nervous system in general, it is reasonable that evidence would have been found in the current study for delays in the development of the central auditory pathways in particular.

## Conclusions

Acquiring human language is (perhaps arguably) the greatest accomplishment of childhood. Although it can appear effortless for children developing in typical fashion, it is in fact a difficult feat requiring that development of many skills proceed “on time” and in a synchronous manner. The purpose of the study reported here was to test two hypotheses related to language acquisition and delay. Hypothesis 1 was that delays in the development of the central auditory pathways can constrain language acquisition, especially of phonological sensitivity as compared to lexicosyntactic knowledge. Hypothesis 2 was that conditions previously demonstrated to hinder language acquisition take their toll in part or wholly through the constraints they impose on the development of the central auditory pathways. Measures of auditory function and language abilities were collected from children spanning the socioeconomic spectrum and varying in gestational age. Results from these children largely supported both hypotheses.
